# Femoral and tibial insert downsizing increases the laxity envelope in TKA

**DOI:** 10.1007/s00167-014-3339-0

**Published:** 2014-10-02

**Authors:** John Kyle P. Mueller, Fred A. Wentorf, Richard E. Moore

**Affiliations:** 1 Zimmer, Inc., P.O. Box 708, 1800 W Center Street, Warsaw, IN 46581-0708 USA; 2 Ada Orthopaedic Clinic, 6500 W Emerald Street, Boise, ID 83704 USA

**Keywords:** Knee, Total knee arthroplasty, Kinematics, Laxity, Cadaver, Robotic testing

## Abstract

**Purpose:**

This study examines the effect of component downsizing in a modern total knee arthroplasty (TKA) system on the laxity envelope of the knee throughout flexion.

**Methods:**

A robotic testing system was utilized to measure laxity envelopes in the implanted knee by in the anterior–posterior (AP), medial–lateral (ML), internal–external (IE) and varus–valgus (VV) directions. Five fresh-frozen cadavers were tested with a modern cruciate retaining TKA implantation, a 1-mm thinner polyethylene insert and a femoral component 2 mm smaller in the AP dimension.

**Results:**

The downsized tibial insert was more lax throughout the flexion arc with up to 2.0 mm more laxity in the AP direction at full extension, a 43.8 % increase over the original implantation. A thinner insert consistently increased laxity throughout the arc of flexion in all degrees of freedom. Downsizing the femoral component resulted in 8.5 mm increase in AP laxity at 90°, a 73.9 % increase. In mid-flexion, downsizing the femur produced similar laxity values to the downsized insert in AP, ML, IE and VV directions.

**Conclusion:**

Downsizing the TKA components had significant effects on laxity throughout flexion. Downsizing a femoral component 2 mm had an equivalent increase in laxity in mid-flexion as downsizing the tibial insert 1 mm. This study quantifies the importance of choosing the appropriate implant component size, having the appropriate size available and the effect of downsizing. The laxity of the implanted knee contributes to how the implant feels to the patient and ultimately the patient’s satisfaction with their new knee.

## Introduction

Total knee arthroplasty (TKA) is a successful treatment for knee pain due to osteoarthritis. However, 25 % of patients are dissatisfied after their TKA surgery [18]. Common complaints include less than desired range of motion (ROM), an unnatural feeling knee, instability and difficulty performing daily activities like ascending or descending stairs [4]. Reduced function has been attributed to laxity in the knee [1, 22], and a recent review of US and Norwegian registries found that between 16 and 20 % of revisions are due to instability [20].

Laxity and functional kinematics have been published in both the intact and implanted knee [1, 5, 8, 9, 12, 14, 15, 24, 25, 35]; however, the effect of downsizing the femoral component and polyethylene insert on the knee’s laxity envelope is not well reported. Surgeons strive to restore function and feel of the unimplanted, healthy knee by matching native boney and soft tissue anatomy with the TKA component options available [19, 26]. The challenge for the surgeon is to create a stable and functional joint that is not too tight or loose [21, 33]. There are several techniques to tune the knee to the desired laxity including adjusting tibial slope, femoral orientation and soft tissue releases [1, 3]. Choosing the correct component size is part of this process and offers another variable for the surgeon to adjust.

If the knee is tight in extension, the surgeon may downsize the tibial insert [21]. If the native femoral anterior–posterior (AP) dimension lies somewhere between available sizes, or the knee is tight in flexion, the surgeon may downsize to a smaller femoral component [13, 21]. Although it is understood that both of these adjustments increase the laxity, the magnitude of this increase has not been investigated in detail and the clinical consequences of this increased laxity are still not well understood [1, 30]. Is there a way to make this balancing act of tuning joint laxity easier for the surgeon and better for the patients?

A traditional TKA system has 3 or 4 mm increments in the AP dimension between femoral sizes and 2 mm between tibial insert thicknesses. To better match the native anatomy and offer more options to better balance the knee, TKA manufacturers have recently gone as far as offering custom implants. Others have increased the number of component sizes available, decreasing the increments between sizes. Some may question whether the costs associated with the extra instrumentation, manufacturing and inventory of these designs outweigh the benefits. This current study quantifies the changes in knee laxity as component sizes are changed.

The testing system utilized in this current study applies repeatable forces and moments to the implanted joint that are meant to replicate the manual laxity tests surgeons use to judge the stability of the knee during TKA surgery. This is important as it quantifies the laxity a surgeon would feel during their intraoperative qualitative evaluation of the knee [11]. Techniques such as adjusting bone cuts to change the tibial slope and femoral orientation, or pie crusting to release ligaments are used to balance the extension and flexion gaps of the knee [21, 26, 28]. Component sizing is also used to tune the knee for optimum stability [13, 21]. This study will investigate the effects of a 1 mm decrease in tibial polyethylene insert thickness or a 2 mm decrease in the femoral component AP dimension. Understanding the effects of component sizing will improve the decision algorithm surgeons use during TKA. We hypothesize that downsizing the femoral or tibial insert sizes 2 and 1 mm, respectively, will affect measured TKA laxity in four degrees of freedom (DOF) throughout the arc of flexion.

## Materials and methods

**Fig. 1:**
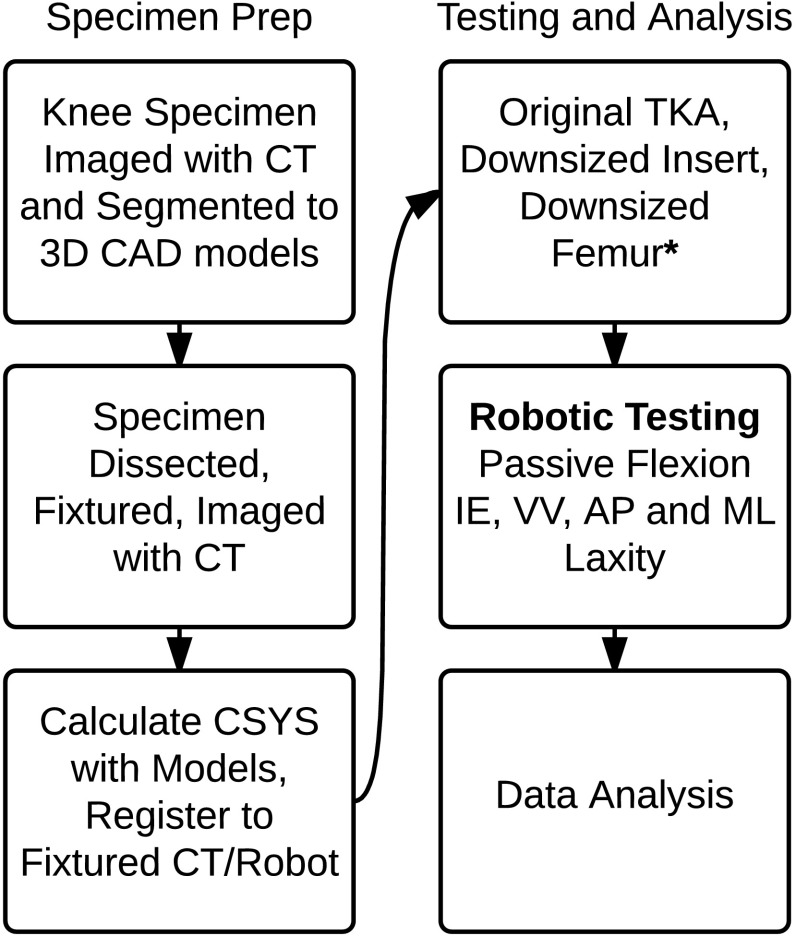
Flow chart describing the study methodology for each specimen. *Robotic testing is performed after Original TKA, Downsized Insert and Downsized Femur implanted states.* CSYS* Anatomically based coordinate system of the knee

**Fig. 2:**
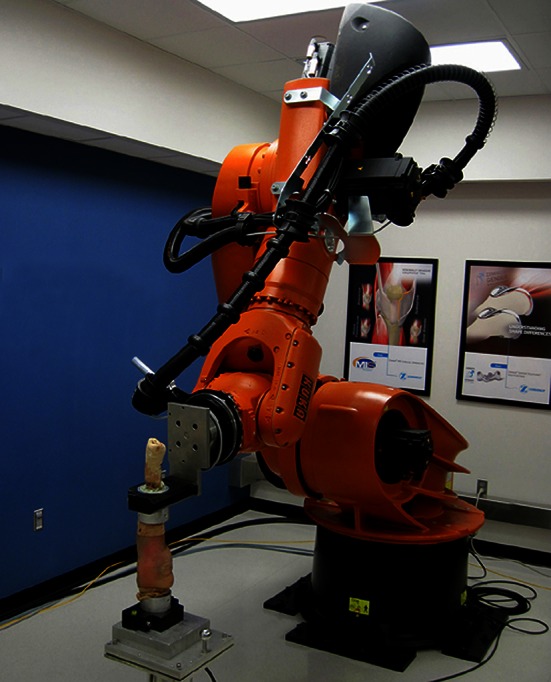
Robot with specimen. The tibia is secured to the robot arm, and the femur is secured to the pedestal which is fixed to the lab floor

The robotic testing method utilized in this study quantified the knee joint laxity by applying repeatable forces and moments in a physiologically defined coordinate system and accurately recording the resulting movement (Fig. 1). The system consisted of a six DOF robot arm (KR500, Kuka Robotics, Ausberg, Germany) and an integrated six DOF load cell (Omega 160 IP65, AMTI, Waltham, MA, USA) (Fig. 2) with average measurement error <1 %. This system applied forces and moments to the knee joint in four physiological directions: anterior–posterior (AP) translation, medial–lateral (ML) translation, varus–valgus (VV) rotation and internal/external (IE) rotation. The overall motion of the femur with respect to the tibia in each physiological direction, which constitutes the laxity envelope, was recorded.

Five fresh-frozen cadaver legs were used in this study. Three-dimensional computer models were constructed (Amira^®^, FEI™, Hillsboro, OR, USA) using computed-tomography (CT) scans (GE VCT 64 slice, GE Healthcare, Wauwatosa, WI, USA) and analyzed to determine an anatomically based coordinate frame. The specimens were prepared by disarticulating the foot and removing tissue to secure aluminum fixtures using bone cement. The fixtures were used to attach the tibia to the robot and secure the femur in a fixed position for testing (Fig. 2). The skin and relevant soft tissues around the knee were kept intact throughout robot testing. A second set of post-fixturing CT scans were registered to the first set of scans (3D Slicer, www.3Dslicer.org) to determine the location of the fixtures relative to the anatomic axis of the knee. The robotic testing system incorporated this information to apply forces and manipulate the specimen relative to the anatomic axis of the knee.

### Original TKA

The specimens were implanted with a modern cruciate retaining TKA (Zimmer™ Persona^®^ Knee, Zimmer, Inc, Warsaw, IN, USA) by an experienced board-certified orthopedic surgeon. A traditional medial arthrotomy was performed splitting the fibers of the distal quad at the interval between the medial 1/3 and the central 1/3. The incision was carried distal along the medial border of the patellae to the medial border of the patellae tendon. The ACL was removed, and the femoral component was implanted using an anterior referencing method. The proximal tibia was cut using an extra medullary guide and confirmed with a plum line from a spacer block. Femoral and Tibial components were selected that best matched the native anatomy, and a tibia insert thickness was chosen to obtain acceptable balancing throughout flexion. The surgeon tested the laxity of the knee by hand with varus valgus stress at 0°, 30°, 60° and 90° of flexion to ensure a balanced knee throughout flexion. No ligament releases were performed during implantation. All components were secured with cement fixation. The knee capsule and skin were closed using sutures and staples, respectively. Post-implantation X-rays were taken to confirm that the implants were placed in the proper alignment. This initial implant state, which would be considered clinically acceptable, was used as the reference condition for the measurements made in this study.

### Downsized insert

The original knee incision was opened exposing the implanted knee joint while avoiding disruption to existing soft tissue. The original polyethylene insert was removed and replaced with a 1 mm thinner polyethylene insert using instruments specific to this knee system. The capsule and skin were sutured closed, and the knee was manually tested for stability before testing.

### Downsized femur

**Fig. 3:**
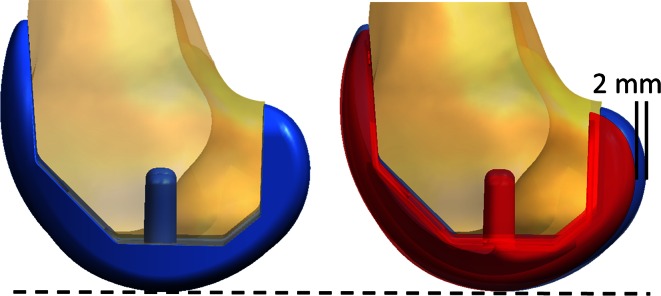
Change in femoral AP dimension from the *Original TKA* to a *Downsized Femur*

The original knee incision was opened. The tibial insert from the *Original TKA* was reinserted, and the femoral component was downsized. In this implant system, the AP dimension differs 2 mm between sizes. As the TKA was implanted using an anterior referencing approach, the anterior and distal cuts of the original femoral component implantation were used as reference. Modifications to the posterior cuts and posterior-distal chamfer cuts were made to accommodate the smaller box of the smaller femur (Fig. 3), taking care not to disrupt any soft tissue structures. Once satisfactory cuts were made, the downsized femoral component was secured using bone cement. The capsule and skin incisions were closed with sutures, and the knee was manually manipulated to ensure stability before testing.

### Passive flexion

The position of the femur with respect to the tibia with low compressive load and no other loads applied to the joint throughout flexion is referred to in this study as the passive flexion path. This is the path the knee would travel guided only by the articulating geometry and soft tissue structures without the influence of large outside forces or moments. The passive flexion path was found using our robotic testing system by applying a 44 N compression force to move the specimen through the ROM while zeroing out all other external forces and moments, except the flexion–extension moment. Zero degrees flexion was used as full extension unless an extension moment threshold of 10 Nm was met before 0°. The passive path was determined from full extension to maximum flexion. The passive flexion tests and subsequent laxity tests were performed on each specimen in all three implanted states described above.

### Laxity testing

**Fig. 4:**
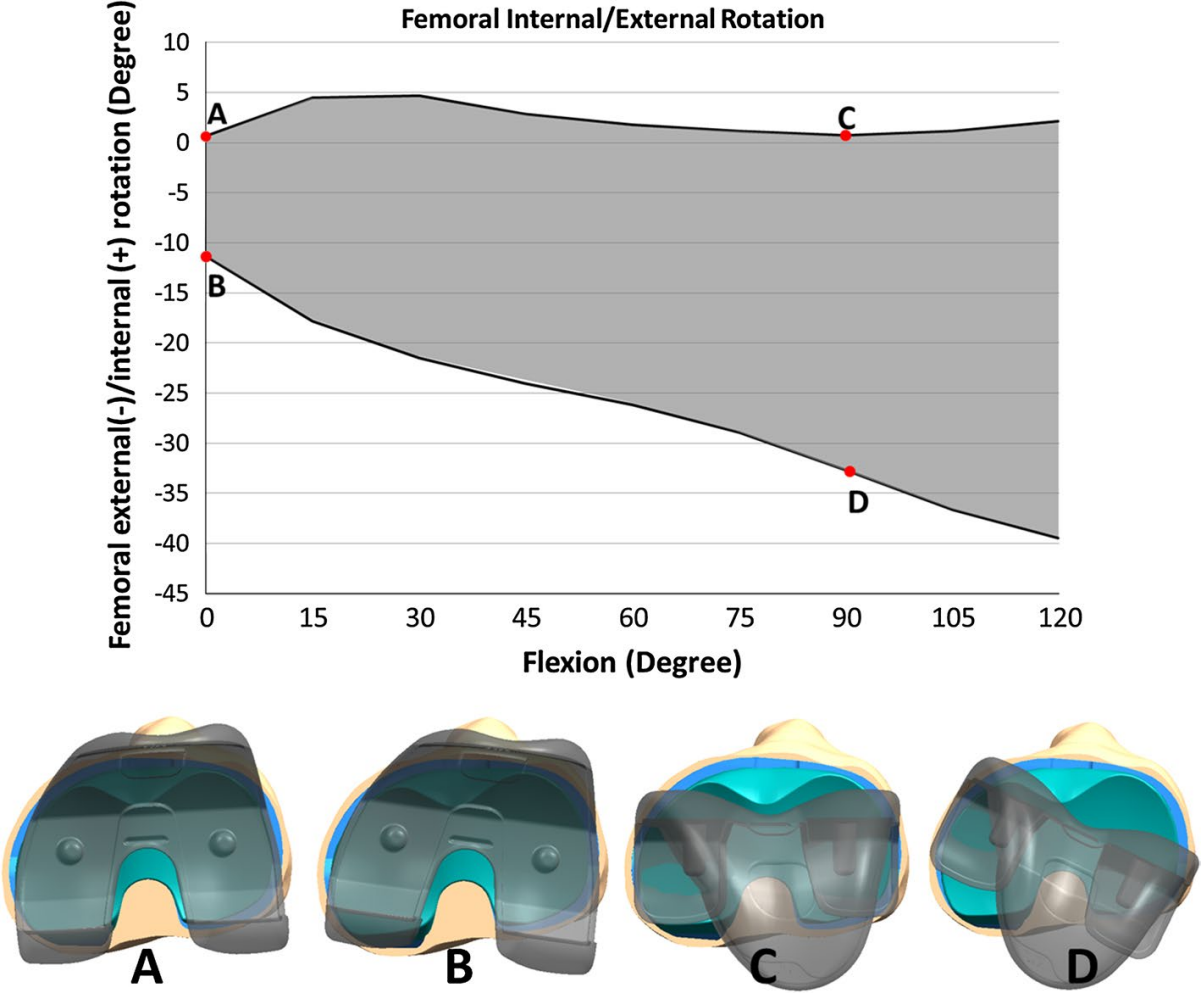
Visual representation of the average internal/external laxity envelope for *Original TKA* at full extension (*a*, *b*) and 90° flexion (*c*, *d*)

For IE laxity evaluations, the knee was initially placed in the passive flexion position of the implanted state at full extension. A torque of 6 Nm was applied about the IE axis in the internal direction and then in the external direction while under 44 N tibiofemoral compressive load. A 44 N load was used to ensure that the articulating geometry remains in contact, but the constraint of the knee is mostly dictated by soft tissue structures. The overall movement from the most internally rotated position to the most externally rotated position constituted the IE laxity envelope at full extension (Fig. 4). The midpoint of the IE laxity envelope was the starting position for the rest of the laxity evaluations at this flexion angle. A similar laxity evaluation was performed in the varus–valgus (VV) rotational direction with 12 Nm torque and the anterior–posterior (AP) and medial–lateral (ML) translational directions with 100 N applied force. These tests were repeated every 15° from full extension to 120° flexion.

Three different laxity metrics were calculated. The average laxity is the average from all specimens in an implanted state at a specific flexion angle. The laxity increase is the average change in laxity from one implant state to another at a specific flexion angle. The percent change is a calculation of the change in laxity at a specific flexion angle from the *Original TKA* to either of the downsized states using the following formula.

$$\% \,{\text{Change}} = \frac{{{\text{Downsized }}\,{\text{laxity}} - {\text{Original TKA }}\,{\text{laxity}}}}{{{\text{Original }}\,{\text{TKA }}\,{\text{laxity}}}} \times 100$$

A positive percent change indicates an increase in laxity from the *Original TKA* to the downsized state. In this study, *Original TKA*, which was the reference condition, is the basis for comparison.

### Statistical analysis

A two-way paired Student’s *t* test was used for each flexion increment to test the null hypothesis that changing the implant state has no effect on laxity and to also test the null hypothesis that the change resulting from each downsized state from the original TKA was not the same. A power analysis revealed five specimens achieve a power of at least 0.8 using sample translational and rotational laxity data at full extension and 90° flexion, except for VV at full extension (power = 0.4) and IE at 90° flexion (power = 0.7). A *p* value <0.05 was deemed statistically significant. Statistical calculations and analyses were performed using Excel^®^ (Microsoft™, Redmond, WA, USA) and Matlab^®^ (The Mathworks™, Natick, MA, USA).

## Results

**Fig. 5:**
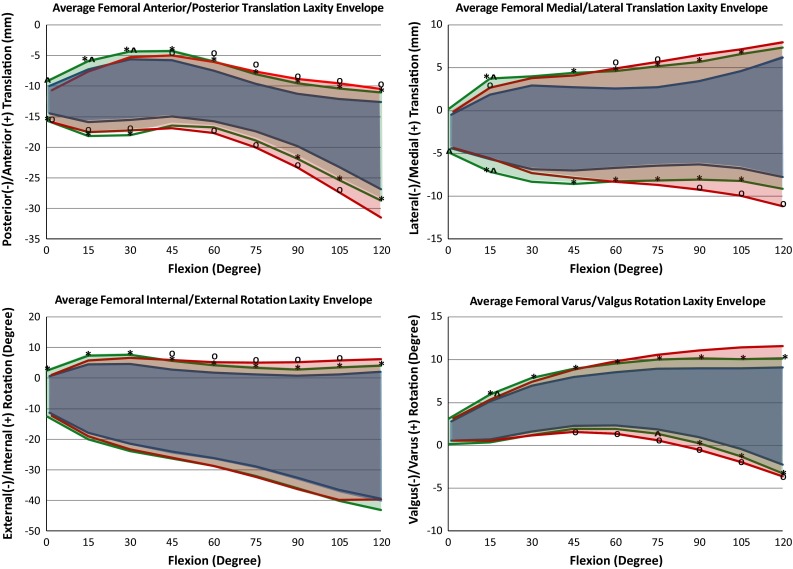
Average envelope of motion for each laxity degree of freedom through flexion for each of the *Original TKA*, *Downsized Insert* and *Downsized Femur* states. *Significant difference (*p* < 0.05) between *Downsized Insert* and *Original TKA*. ^O^Significant difference (*p* < 0.05) between *Downsized Femur* and *Original TKA*. ^Significant difference (*p* < 0.05) between *Downsized Insert* and *Downsized Femur*

**Fig. 6:**
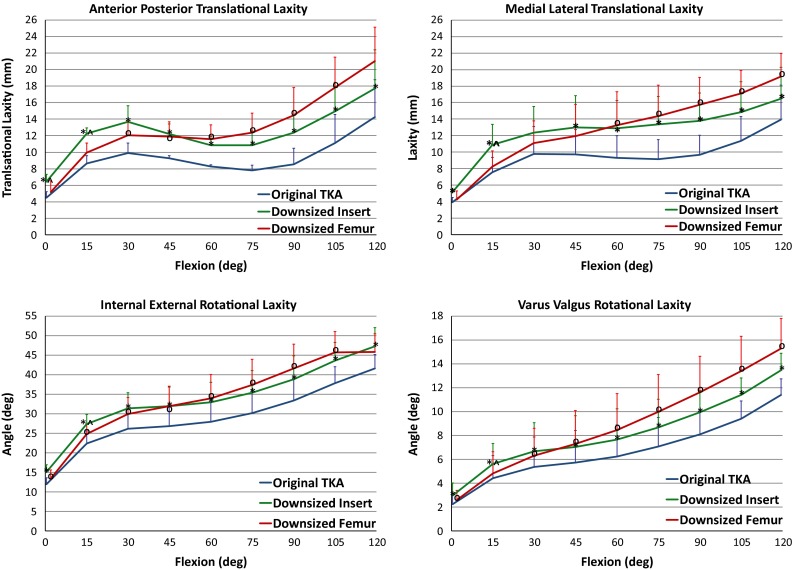
Laxity plots for each degree of freedom through flexion. Laxity is the total motion within the laxity envelope at each flexion increment. *Significant difference (*p* < 0.05) between *Downsized Insert* and *Originial TKA*. ^O^Significant difference (*p* < 0.05) between *Downsized Femur* and *Original TKA*. ^Significant difference (*p* < 0.05) between *Downsized Insert* and *Downsized Femur*

Downsizing the insert and downsizing the femur both increased the measured laxity of the implanted TKA. Similar trends spanned the four laxity modes as the knees progressed through flexion. All three states averaged the least amount of laxity at full extension and the greatest amount of laxity at 120° flexion in all four DOF tested (Figs. 5, 6). The laxity curves as a result of downsizing the tibial insert 1 mm were similar in shape to the *Original TKA* (Fig. 6) as there was a consistent increase in laxity through the whole arc of flexion. The 2 mm change in femoral AP dimension had a minimal effect in extension but increased laxity compared to the *Original TKA* as the knee progressed into flexion.

### Early flexion (0°–30°)

Four of five specimens in the *Original TKA* state reached the 0° limit for full extension. One specimen was flexed 6° in the *Original TKA* but after conversion to the *Downsized Insert* was flexed 3° at the 10 Nm extension moment, achieving three additional degrees of extension.

The laxities more than doubled from full extension to 30° flexion over most DOF for the *Original TKA* and both downsized states (Figs. 5, 6). The *Downsized Insert* was significantly more lax than the *Original TKA* in all four laxity directions throughout early flexion (*p* < 0.05). There were significant differences between the *Downsized Femur* and *Original TKA* in early flexion; however, the *Downsized Femur* was tighter than the *Downsized Insert* in all four DOF (*p* < 0.05) as would be expected (Fig. 6).

**Fig. 7:**
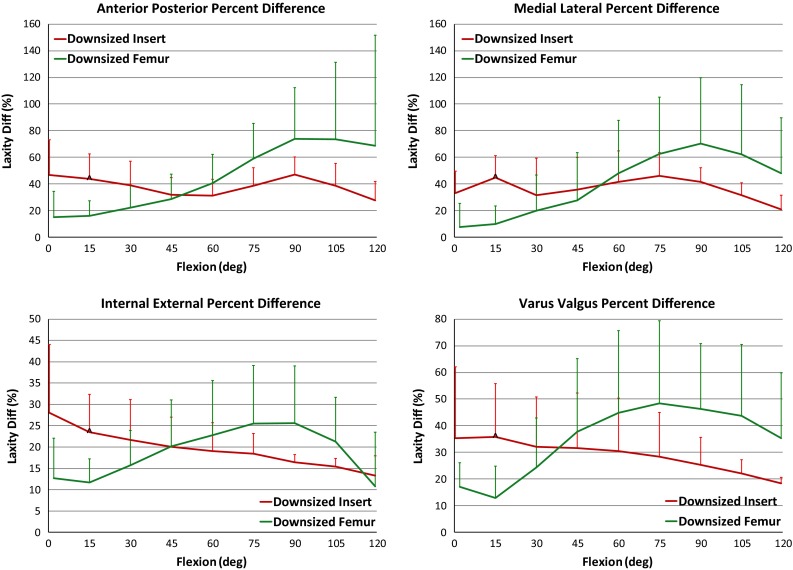
Plot of percent change in laxity from the *Original TKA* to the respective downsized states. ^A difference in percent change between the downsized states (*p* < 0.05)

Downsizing the tibial insert 1 mm resulted in a greater increase in average laxity compared to downsizing the femur, but this was only significant at 15° flexion across all DOF (AP *p* = 0.017, ML *p* = 0.016, IE *p* = 0.016, VV *p* = 0.023). In the AP direction at full extension, there was a 2.0 mm (43.8 %) and 0.7 mm (15.9 %) increase in laxity after converting to *Downsized Insert* and *Downsized Femur*, respectively (*p* = 0.045) (Figs. 6, 7). At 15° flexion in VV, there was a 1.2° (35.9 %) and 0.4° (12.9 %) increase in laxity from the *Original TKA* to *Downsized Insert* and *Downsized Femur*, respectively (*p* = 0.023).

### Mid-flexion (30°–60°)

The laxity changes within the three implanted states from the start to end of mid-flexion were relatively small compared to early flexion (Figs. 5, 6). The two downsized states were significantly different than the *Original TKA* at 30°, 45° and 60° flexion in all laxity directions (*p* < 0.05) (Fig. 6). However, there was not a significant difference between the downsized states. For example, the average VV laxity at 45° flexion was 5.7°, 7.0° and 7.3° for the *Original TKA*, *Downsized Insert* and *Downsized Femur*, respectively. This represents a 31.7 and 37.7 % change from the *Original TKA* (Fig. 7). The *Downsized Insert* saw an average percent change of 31.9 % and the *Downsized Femur* of 28.7 % compared to the *Original TKA* in the AP direction at 45° flexion (*p* = 0.730) (Fig. 7). The similarity in laxity values and laxity change indicates downsizing the femoral component by 2 mm has a similar effect in mid-flexion as decreasing the tibial insert thickness 1 mm.

### Late flexion (60°–120°)

There was a relatively large laxity increase into deeper flexion in the original and downsized states (Figs. 5, 6). The average laxities of the two downsized states were significantly different from the *Original TKA* in all laxity modes through most of later flexion (*p* < 0.05) (Fig. 6). At 90° flexion, the AP laxity for the *Original TKA* was 8.5 mm and the VV laxity was 8.1°, compared to the Downsized Insert at 12.4 mm (*p* = 0.0005) and 9.9° (*p* = 0.0004) and the Downsized Femur at 14.5 mm (*p* = 0.012) and 11.6° (*p* = 0.020), respectively (Figs. 5, 6).

The *Downsized Insert* percent increase from the *Original TKA* ranged from 16.5 % in IE to 41.6 % in ML at 90° flexion (Fig. 7). The average percent increase in laxity from *Original TKA* to *Downsized Femur* at 90° ranged from 25.6 % in IE to 73.9 % in AP. Although not reflected in the percent change calculations and not a significant difference (n.s.), the change in laxity from the *Original TKA* to a 2 mm smaller femur nearly doubled the increase in laxity from the *Original TKA* to the 1 mm thinner insert at 120° flexion in all DOF except IE.

## Discussion

The most important finding in this study is the significant effect relatively small changes in TKA dimensions have on the laxity of the knee in four DOF throughout the arc of flexion.

Downsizing the tibial insert 1 mm increased laxity up to 43.8 %. The main stabilizers in these low compressive load tests are the tibiofemoral articulating geometry and ligamentous soft tissues [29]. Changes in TKA component geometry or placement have been shown to change the length or tension of ligaments [9, 28]. Slight reductions in ligament length can significantly reduce ligament tension [32] and in turn increase the laxity of the joint. This is supported by Walker et al. [28] in a study of knee balancing using instrumented tibial trials. Downsizing the femoral component had minimal effect on laxity in full extension as the distal resection did not change; therefore, the joint space in extension was not affected. However, there was a significant laxity increase in flexion up to 73.9 %. Since this was an anterior referencing technique, a significant laxity increase at 90° would be expected as the AP dimension of the femur is aligned with the direction of the collateral ligaments. The change decreases the posterior condylar offset (PCO). The clinical effects of decreasing the PCO are still controversial [2, 10, 17, 30, 33]. It is not surprising based on the linear nature of ligament stiffness [32] how much laxity increases in later flexion after changing 2 mm compared to 1 mm. A revealing finding is that the laxities of the *Downsized Femur* and *Downsized Insert* were similar in mid-flexion. This study shows that reducing the AP dimension of the femoral component by 2 mm had a similar or greater effect throughout flexion as downsizing the tibial insert thickness by 1 mm. Although the femoral change is in the AP direction, this change affects the joint space earlier than 90° flexion. These findings indicate undersizing components may contribute to instability in mid-flexion and could be added to the list of possible causes [24, 27, 31, 34].

This study has strengths and limitations that should be considered. Laxity evaluations are clinically relevant in that surgeons use this intraoperative assessment to guide their implantation technique. Cadaveric laxity evaluations have been shown to correlate well with laxity evaluations performed on live subjects [16]. Thus, the findings from this study should correlate well with the clinical arena.

This study has several limitations including examining one level of downsizing. An additional level of downsizing or upsizing the components would provide a trend of the effect of adjusting component size on laxity. Also, cadaveric models only represent surgery immediately post-operatively. Additionally, testing more than five specimens would increase the power of this study. However, these are matched comparisons, the study was well powered in most DOF and the number compares to similar cadaveric studies [5, 14, 15, 28]. The VV direction at full extension and IE at 90° is lower in power than the other DOF, and this should be noted. Another limitation is that the experiments were carried out under a small compressive load. While this does not represent functional load-bearing conditions, it does represent the situation at the time of TKA surgery during the balancing process.

Increases in knee joint laxity can contribute to the feeling of instability, joint pain, increased implant wear and affect daily activities [21]. In a cruciate retaining design, downsizing will affect the engagement of the PCL and potentially limit posterior rollback during flexion [5, 23]. Increased varus/valgus laxity would allow femoral condylar lift-off [6, 7], which is unnatural for the patient and increases stress on the polyethylene insert. Larger motion in the joint could lead to synovial impingement or retinacular strain resulting in pain, and potentially inflammation and joint effusion. In a stable knee joint, the ligaments keep the femur in an appropriate position relative to the tibia [28]. If the femur moves to an unnatural position while unloaded, a correction is needed during initiation of stance phase of walking, descending stairs or more strenuous activities requiring change of direction. Although subtle, the patient could sense a delayed response time or instability contributing to the unnatural feeling knee.

## Conclusions

Surgeons have several techniques during TKA to restore satisfactory knee function, stability and feeling to the patient, one of which is component sizing. This study shows the importance of choosing the appropriate implant component size, having the appropriate size available and the effect of downsizing. A relatively small decrease in insert and femoral component size increases laxity throughout the arc of flexion up to 43.8 % and 72.9 %, respectively, compared to the original component implantation. These findings also reveal that a 2 mm downsizing of the femoral AP dimension increases laxity not only in flexion, but were shown to increase laxity in the mid-flexion range equivalent to downsizing the polyethylene insert by 1 mm.
